# BiSpark: a Spark-based highly scalable aligner for bisulfite sequencing data

**DOI:** 10.1186/s12859-018-2498-2

**Published:** 2018-12-10

**Authors:** Seokjun Soe, Yoonjae Park, Heejoon Chae

**Affiliations:** 1 Department of Computer Science and Engineering, Seoul National University, Seoul, Republic of Korea; 2 Seoul National University, Seoul, Republic of Korea; 3 Division of Computer Science, Sookmyung Women’s University, Seoul, Republic of Korea

**Keywords:** DNA methylation, Bisulfite sequencing, Alignment, Apache Spark

## Abstract

**Background:**

Bisulfite sequencing is one of the major high-resolution DNA methylation measurement method. Due to the selective nucleotide conversion on unmethylated cytosines after treatment with sodium bisulfite, processing bisulfite-treated sequencing reads requires additional steps which need high computational demands. However, a dearth of efficient aligner that is designed for bisulfite-treated sequencing becomes a bottleneck of large-scale DNA methylome analyses.

**Results:**

In this study, we present a highly scalable, efficient, and load-balanced bisulfite aligner, BiSpark, which is designed for processing large volumes of bisulfite sequencing data. We implemented the BiSpark algorithm over the Apache Spark, a memory optimized distributed data processing framework, to achieve the maximum data parallel efficiency. The BiSpark algorithm is designed to support redistribution of imbalanced data to minimize delays on large-scale distributed environment.

**Conclusions:**

Experimental results on methylome datasets show that BiSpark significantly outperforms other state-of-the-art bisulfite sequencing aligners in terms of alignment speed and scalability with respect to dataset size and a number of computing nodes while providing highly consistent and comparable mapping results.

**Availability:**

The implementation of BiSpark software package and source code is available at https://github.com/bhi-kimlab/BiSpark/.

## Background

DNA methylation plays a critical role in gene regulation process. It is well-known that promoter methylation causes suppression of down stream gene transcription, and abnormal DNA methylation status of diseases-associated genes such as tumor suppressor genes or oncogenes are often considered as biomarkers of the diseases. In addition, promoter methylation especially at the transcription factor binding sites (TFBS) changes the affinity of TF binding result in abnormal expression of downstream genes. Thus, measuring DNA methylation level now becomes one of the most desirable follow-up studies for transcriptome analysis. Various measurement methods for DNA methylation were previously introduced. Illuminaś Infinium HumanMethylation 27K, 450K, and MethylationEPIC (850K) BeadChip array cost-efficiently interrogate the methylation status of certain number of CpG sites and non-CpG sites across the genome at single-nucleotide resolution depending on their coverages. Methylated DNA immunoprecipitation-sequencing (MeDIP-seq) [1] isolates methylated DNA fragments via antibodies followed by massively parallelized sequencing. Methyl-Binding Domain sequencing (MBD-seq) utilizes an affinity between MBD protein and methyl-CpG. These enriched DNA methylation measurement methods have been used to estimate genome-wide methylation level estimation.

Bisulfite sequencing is one of the most well-known methylation measurement techniques to determine methylation pattern in single base-pair resolution. Bisulfite sequencing utilizes the characteristic of differential nucleotide conversion between methylated and unmethylated nucleotides under the bisulfite treatment. By utilizing bisulfite treatment technique, whole genome bisulfite sequencing (WGBS) can measure DNA methylation statuses of the entire genome. Due to the nucleotide conversion caused by bisulfite treatment, reads from the bisulfite sequencing have higher mismatch ratio than whole genome sequencing. As a result, bisulfite-treated reads requires a specialized alignment algorithm to correctly estimate the methylation levels. Compared to the WGBS measuring genome-wide DNA methylation status, Reduced representation bisulfite sequencing (RRBS) [2] selects 1% of the genomic regions that are considered as key regions related to gene transcriptional process such as promoters. RRBS uses restriction enzyme to reduce genome complexity followed by subsequent bisulfite treatment. Due to the high cost of measuring the whole genome DNA methylation status, the cost-efficient RRBS technique becomes a popular alternative method measuring the DNA methylation in single-nucleotide resolution.

In order to handle bisulfite-treated reads, various approaches have been proposed. Because of the nucleotide conversion of un-methylated cytosine (umC) to thymine by the bisulfite treatment, sequenced reads from bisulfite sequencing require to discriminating whether the Ts in the reads come from original DNA nucleotide or from converted nucleotide (umC). Bismark [3] and BSSeeker [4] use the ‘three-letter’ approach [5] to determine the origin of bisulfite-treated nucleotides. In ‘three-letter’ approach, all cytosines in reference genome and bisulfite-treated reads are converted to thymines in order to reduce the ambiguity of thymines. General DNA read alignment algorithm is used to find the best mapping position of the read, and then methylation levels are measured from the unconverted reference genome and reads. BRAT-BW [6] adopts this ‘three-letter’ approach with the multi-seed and uses FM-index to achieve higher efficiency and lower memory footprint respectively. On the other hand, BSMAP [7] and RMAP [8] utilize wildcard concept to map the ambiguous bisulfite-treated reads. In wildcard approach, both cytosines and thymines are allowed to map on cytosines in the reference genome. A heuristic approach was also introduced to improve the mapping sensitivity of bisulfite-treated reads. Pash[9] employs collating k-mer matches with neighboring k diagonals and applies a heuristic alignment.

Among these several approaches of mapping bisulfite-treated reads, the ‘three-letter’ algorithm is the most widely used because it has showed better alignment performance in various perspectives [5]. However, even the aligners using the ‘three-letter’ algorithm shows relatively better performance in terms of mapping accuracy, they are still suffer from high computational demands because in the ’three-letter’ algorithm, the aligning step requires to process at most four times more volumes of data (two times more for each directional library reads) to correctly estimate the DNA methylation level (discrimination between original thymine and thymine converted from umC). Thus, measuring DNA methylation level by widely-used ‘three-letter’ approach is still considered as one of the significant bottlenecks of entire methylome data analysis. Even though some aligners, such as Bismark and BS-Seeker2, offer multi-core parallel processing to alleviate this shortcoming of the ’three-letter’ approach, they are still not well scaled-up enough and limited within a single node capacity of computational resources. Besides, since increasing the computing resources, such as CPU/cores and memory within a single large computing server, called scale-up, rapidly drops the cost-effectiveness, it has been widely researched to achieve higher performance by using a cluster of computers instead, called scale-out. Considering financial factors, the scale-out approach can be more affordable to users and well-designed scale-out approach usually shows better scalability than scale-up approach [10]. As a result, in order to overcome the limitation of a single node scale-up approach, distributed system, such as cloud environment, has been considered as an alternative solution to the multi-core model.

The distributed system approach was first adopted to map DNA sequences and related data-intensive processing tasks. Cloudburst [11] and CloudAligner [12] were introduced to improve the mapping performance by using MapReduce framework [13]. They are executed parallelly on multiple nodes based on Hadoop framework [14] and achieve efficient large-scale alignment on the distributed system. Crossbow [15] is an another application that utilizes the multi-node approach to resolve the performance problem of alignment and SNP calling. Crossbow is an analysis software pipeline designed to run in the cloud environment (especially on the Amazon Elastic MapReduce [16]) and thus allows dynamic allocation of computing resources. SparkBWA [17] adopts recently introduced Apache Spark framework [18], a memory-optimized software framework designed for large-scale data processing on distributed cluster of computers, accelerating BWA aligner [19] on the multiple computing nodes.

There exist aligners that adopt the multi-node concept for processing the bisulfite-treated sequencing datasets. The CloudAligner provides an option for handling the bisulfite-treated reads within their algorithm. Bison [20] utilizes MPI (Message Passing Interface) library [21] to process bisulfite sequencing data over the cluster. However, these algorithms are still suffering from either lack of functionalities and poor performance due to originally being designed for a general purpose aligner, or not scaled enough especially in the large volumes of methylome analysis.

In order to overcome such drawbacks, we developed the *BiSpark* algorithm, a highly scalable, efficient, and load-balanced bisulfite aligner that utilizes distributed environment to significantly improve aligning speed and scalability while keeping reasonable mappability, precision, sensitivity, and accuracy. The *BiSpark* algorithm is designed to fully utilize the benefits of recently introduced Apache Spark distributed framework. In the *BiSpark* algorithm, we designed a well-parallelized ‘three-letter’ mapping algorithm fitting on Spark framework, resulting in scaling out almost linearly. In addition, implemented a highly-optimized load-balancing algorithm in the *BiSpark* provides re-distributing data almost evenly across the cluster nodes, achieving better scalability on a large-scale cluster.

## Implementation

We completely redesigned and implemented ‘three-letter’ algorithm suitable for the distributed Apache Spark environment. The basic concept of ‘three-letter’ algorithm was adopted from BSSeeker2 [22], while we designed the parallelized version of ‘three-letter’ algorithm to fit into RDD (Resilient Distributed Datasets) and key-value concepts [23] of the Spark framework.

We also integrated HDFS (Hadoop Distributed File System) [24] to provide centralized data management, which makes *BiSpark* to efficiently handle shared data among cluster while user need not bother with distributing data. Following is implementation details of the *BiSpark* algorithm.

### Genome preparation

The ‘three-letter’ algorithm essentially requires transforming reference genomes into customized reference genomes that consist of only three nucleotides, and this needs four types of genome transformations (all cytosine to thymine (CT) conversion and guanine to adenine (GA) conversion for each Watson (W) and Crick (C) strand, resulting in W-GA, W-CT, C-GA, C-CT conversion). In *BiSpark*, all four reference genome conversion and indexing are performed in the master node first and moved to HDFS for multi-node sharing.

### Analysis workflow

**Fig. 1:**
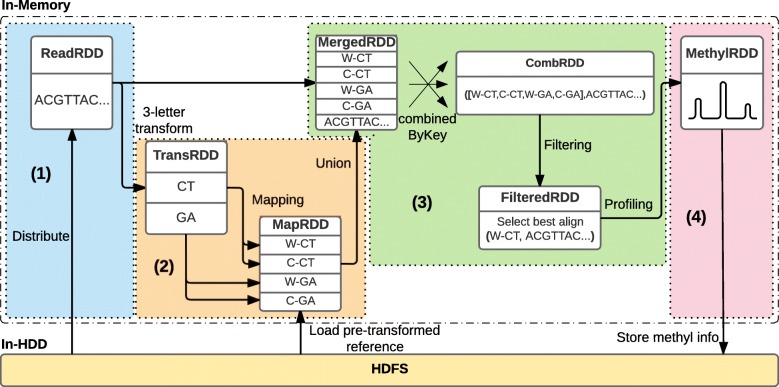
Analysis workflow within *BiSpark* consists of 4 processing phases: (1) Distributing the reads into key-value pairs, (2) Transforming reads into ‘three-letter’ reads and mapping to transformed reference genome, (3) Aggregating mapping results and filtering ambiguous reads, and (4) Profiling the methylation information for each read. The figure depicts the case when library of input data is a non-directional

The workflow of *BiSpark* consists of four major parts (Fig. 1): (1) converting data into key-value RDD structure, (2) transforming reads into ‘three-letter’ reads and mapping to customized reference genome, (3) finding best alignment and filtering, and (4) profiling methylation information for each read. Following is the details of each *BiSpark* analysis phases.

#### Phase 1: converting to key-value RDD structure

At initial stage, the *BiSpark* accepts raw sequencing data files, FASTQ/A format, as inputs and converts them into list of key-value structured tuples; the first column is a read identifier (key) and the second column is a read sequence (value). At the same time, the *BiSpark* stores these tuples into the RDD blocks, named as readRDD, which is the basic data structure used in Spark framework. Since the RDDs are partitioned and placed over memories of cluster nodes, the *BiSpark* could distribute input data across the cluster as well as keep them in main memory, which can reduce I/O latency if the data is re-used. As a result, the *BiSpark* algorithm could minimize physical disk access, resulting in a significant speed up during follow-up data manipulation phases.

#### Phase 2: ‘three-letter’ transforming and mapping

Mapping the bisulfite-treated sequencing data, which has innate uncertainty, requires additional data manipulation steps. In order to handle this on the distributed environment, the *BiSpark* transforms readRDDs to transRDD which consists of <read id, transformed read sequence > tuples. These transRDDs are subcategorized into CTtransRDD (cytosine to thymine conversion) and GAtransRDD (guanine to adenine conversion), which reduces uncertainties of bisulfite-treated reads from each Watson and Crick strand respectively.

Once the transRDDs are created, the *BiSpark* aligns each of the transRDDs to ‘three-letter’ customized reference genomes. We adopted *Bowtie2* for mapping reads to reference genome, known as one of the best DNA sequence aligner [22]. During the mapping process, the *BiSpark* aligns each transRDD loaded on the memory of each distributed node, and generates another list of tuples, called mapRDD. By utilizing quality information, poor reads are discarded. These mapRDDs contains information of read-id with alignment results including general alignment information, such as number of mismatches and genomic coordinates, as well as specialized information, such as conversion type of transRDD. These mapRDDs have read id as the key while having alignment result including the number of mismatches and genomic coordinates and additional information, such as a conversion type of transRDD. The mapRDDs are subcategorized into W-CTmapRDD, W-GAmapRDD, C-CTmapRDD and C-GAmapRDD depending on the alignment pairs between the transRDDs and the customized reference genomes. At the end of aliment process, the *BiSpark* keeps all the mapRDDs within the main memory so as to be accessed rapidly in following steps.

#### Phase 3: finding the best alignment

Data transfer between nodes is one of the biggest obstacles in distributed data processing. In the ‘three-letter’ algorithm, two converted reads (CT, GA) are generated from a single read, and mapping these reads creates four different alignment results (W-CT, W-GA, C-CT, and C-GA). In order to handle the ambiguity caused by bisulfite-treatment, the next step of the analysis is figuring out the best alignment among these results. In a distributed system, these four different alignment results are dispersed across multiple nodes, and to find the best sort, the alignment results with the same key need to be rearranged to be located on the same node. This transfer and redistribution of the data between nodes, called ‘shuffling’, need to be performed per every single read, and thus it is one of the most time-consuming part of the distributed algorithm. In general, how to minimize the number of shuffling phases is a major issue for designing a distributed algorithm and has significant impact on the performance.

To alleviate the issue of ‘three-letter’ algorithm implemented in distributed system, we designed each mapRDD to use the same partition algorithm and to be divided into the same number of partitions. Then, if we applied context-level union function, offered by Spark, the shuffling does not occur while all mapRDDs are merged into a single RDD due to the design of Spark framework. As a result, the distributed version of ‘three-letter’ algorithm implemented within the *BiSpark* could significantly reduce the processing time. Finally, the aggregated alignment results are combined by read id, resulting in a single RDD, called combRDD, whose value is a list of mapping results.

The ‘three-letter’ transformation reduces mismatches of alignment, but increases the probability of the false-positive alignments. To solve this known issue, most ‘three-letter’ mapping algorithms have strong restrictions to determine if the mapping result is valid [3, 4, 22]. In the *BiSpark* algorithm, the best alignment among the results is the alignment that has the uniquely least number of mismatches. If multiple alignments have the same smallest number of mismatches, the read and corresponding alignments are considered ambiguous, thus discarded. Moreover, the *BiSpark* also supports a user-defined mismatch cutoff to adjust the intensity of the restriction depending on the situation. All results not satisfying these conditions are discarded, resulting in the filteredRDD. Through these steps, the *BiSpark* could keep high mappability (details in “Mapping quality evaluation” section).

#### Phase 4: methylation profiling

In ‘three-letter’ algorithm, read sequence, mapping information, and original reference genome sequence are required to estimate methylation status at each site. In distributed environment, gathering all these information together from the multiple nodes requires multiple shuffling operations, which is time-consuming. To minimize multi-node data transfer during the methylation calling phase, we combined the read sequence and mapping information from the readRDD and mapRDD respectively, and designed new RDD, called mergedRDD. In this way, although the size of each tuple is slightly increased, the information of read sequence could be delivered to filteredRDD with mapping information which means the *BiSpark* could avoid additional shuffling operations. In addition, since the original reference genome sequence also required to be staged to the multi-nodes, the *BiSpark* minimize the reference staging time via broadcasting it by utilizing shared variable functionality of the Spark framework allowing direct access to the reference genome sequence from the multi-nodes. Based on these optimized implementation, the *BiSpark* could achieve significant performance gain compared to other algorithms (see details in “Scalability evaluation to data size” and “Scalability evaluation to cluster size” sections). Finally, methylRDD has the methylation information, estimated by comparing filteredRDD with the original reference genome sequence, as the value. The methylRDD is finally converted to SAM [25] format and stored in HDFS.

### Load balancing

Single node delay due to unbalanced data distribution in distributed data processing makes the entire cluster wait. As a result, load balancing over the nodes of the cluster is one of the most important issues when designing a parallel algorithm.

While designing the ‘three-letter’ algorithm in distributed environment, we investigated the data imbalance at each phase and found that there exist two possible bottleneck points. The first point is where HDFS reads sequence data. When Spark reads data from HDFS, it creates partitions based on the number of chunks in HDFS, not the number of executers, so each Spark executor is assigned different size of input data. Another imbalance can be found after the phrase of finding the best alignment followed by filtering. This is because the ratio of valid alignment would be different for each partition.

In order to prevent the delays caused by imbalances, the *BiSpark* applied hash partitioning algorithm. Even though hash partitioning does not ensure perfectly balanced partitions, the data would be approximately well distributed because of the hash function. At each of the data imbalance points, the *BiSpark* utilizes *portable_hash* function, supported by Spark framework, to determine which partition the data should be placed. By re-partitioning data with the applied hash function, implementation of the ‘three-letter’ algorithm in the *BiSpark* could expect the well-distributed data across the multiple nodes. Although introducing extra partitioning improves parallel efficiency, it requires additional shuffling operation, which takes additional processing time. Considering the trade-off, the *BiSpark* offers the load balancing functionality as an option, enabling users to select proper mode depending on the cluster size. For more details of the performance gain from the implemented load balancing within the *BiSpark* algorithm, see “Scalability evaluation to data size” and “Scalability evaluation to cluster size” sections.

## Experiment

### Bisulfite-treated methylome data

For our experimental studies, we evaluated the algorithms on both simulation data sets and real-life data sets. Simulation data was generated by Sherman [26] (bisulfite-treated Read FastQ Simulator), already used by previous studies [20], setting up with human chromosome 1, read length to 95bp, and the number of reads to 1,000,000. We prepared three datasets with error ratio in 0%, 1%, and 2% for accuracy evaluation.

Real data set is a whole genome bisulfite sequencing (WGBS) dataset obtained from Gene Expression Omnibus (GEO) repository whose series accession number is GSE80911 [27]. The sequencing data was measured by Illumina HiSeq 2500 in 95bp length. For the performance evaluation, we cut the entire data out to create the various size of testing data sets. During aligning process for performance evaluation, we used human reference genome (ver. Build 37, hg19). The statistics of the data sets used in our experiments are summarized in Table 1.

**Table 1:** Experimental data for performance evaluation

Data set	Tailored data size	# of reads	Description
Simulation data	122MB	1,000,000	Simulation set with 0% error
	122MB	1,000,000	Simulation set with 1% error
	122MB	1,000,000	Simulation set with 2% error
GEO WGBS data (GSE80911)	1.6GB	10,000,000	10 million reads real data set
	7.9GB	50,000,000	50 million reads real data set
	16GB	100,000,000	100 million reads real data set
	32GB	200,000,000	200 million reads real data set
Reference genome	Build 37, hg19		

Simulation data sets are generated by Sherman [26] with various error rates (0%, 1% and 2% respectively) where the error rate is a mean error rate per bp whereby the error curve follows an exponential decay model. Each test data sets are tailored from original WGBS data based on number of reads

### Experimental design

We empirically evaluated performance of the *BiSpark* with existing state-of-art bisulfite aligning methods. We first compared the *BiSpark* to aligners, CloudAligner and Bison, implemented based on distributed environment. CloudAligner is a general short-read DNA aligner running on the Hadoop MapReduce framework that includes bisulfite-treated read alignment function while Bison a recently introduced distributed aligner specifically designed for processing bisulfite-treated short reads via utilizing MPI library. The performance of algorithms is tested in terms of scaling out with respect to data size and cluster size over the cluster of multiple nodes. We also compared the *BiSpark* to a single-node but multi-core parallel bisulfite aligner. We selected Bismark for single server aligner since Bismark has been evaluated as the best performance bisulfite aligner without losing the sensitivity [5, 28] within the single-node parallelization category.

We first evaluated four metrics including mappability, precision, sensitivity and accuracy from simulation data. Unlike real data, simulation data reports the original position of generated read, which enables us to measure the metrics. The details of how we calculated metrics are described below.

$$\begin{array}{lcl} TP & = & \text{number of correctly mapped reads} \\ FP & = & \text{number of incorrectly mapped reads} \\ FN & = & \text{number of unmapped reads} \\ mappability & = & \frac{\text{number of mapped reads}}{\text{number of all reads}} \\ precision & = & \frac{TP}{TP+FP} \\ sensitivity & = & \frac{TP}{TP+FN} \\ accuracy & = & \frac{TP}{TP+FP+FN} \\ \end{array} $$

The more the error in reads, the harder the reads are correctly mapped. Therefore, we measured metrics while increasing error ratio.

We also evaluated the scalabilities of the aligners to data size and number of nodes of the cluster with real data. To compare *BiSpark* with existing aligners, we built 3 clusters which consist of 10, 20, and 40 computing nodes respectively while each of cluster has one additional master node. We also prepared a single server with 24 cores to measure the performance and indirectly compare with non-distributed aligner, Bismark. Our constructed testing environment is summarized in Table 2.

**Table 2:** Testbed for performance evaluation

System/framework	description	version
Master	1 master node of cluster	CPU: 2.2GHz
	(Intel Xeon E5-2407)	Memory: 8GB
Slaves	{10,20,40} slave nodes of cluster	CPU: 3.3GHz
	(Intel i3-3220)	Memory: 8GB
Single server	24 core single server	CPU: 2.6GHz
	(Intel Xeon X5650)	Memory: 94GB
Apache Hadoop	Distributed file system	v2.6.0
Apache Spark	Data processing framework	v1.6.0
Bowtie2	General short read aligner	v2.2.9
CloudAligner	Bisulfite aligner on cluster	v1.8
Bison	Bisulfite aligner on cluster	v0.3.3
Bismark	Bisulfite aligner on single machine	v0.18.1

We denoted *BiSpark* without additional load balancing implementation as *BiSpark*-plain while *BiSpark* with load balancing is denoted as *BiSpark*-balance. For all aligners, there are some pre-processes including transforming and indexing reference genome, distributing input file and changing the format of the input file. Because pre-processing is alinger-specific and can be reused continuously after running once, we exclude pre-processing time when measuring elapsed time. For the reference genome, we used chromosome 1 of human genome because the CloudAligner can only process single chromosome at a time. We tested all aligners in non-directional library mode. When executing Bison, we used 9, 21 and 41 nodes for the 10-cluster, 20-cluster, and 40-cluster experiments respectively. This is because, in the Bison aligner, there exist a restriction on the setting of a number of nodes that allows only 4((*N*−1)/4)+1 nodes if there are *N* nodes.

## Results

### Mapping quality evaluation

Table 3 shows mappability, precision, sensitivity and accuracy of aligners for each simulation data set. The results of CloudAligner are excluded from the table since it fails to create correct methylation profiles over the simulation datasets. From the evaluation results, the *BiSpark* shows the best performance on all four metrics with the 0% error dataset. In addition, as the error rate increases, the *BiSpark* still shows the best performance on mappability and sensitivity, and reasonably high precision. From these evaluations, we could confirm that the *BiSpark* algorithm is accurate and robust enough to the errors.

**Table 3:** Mappability, precision, sensitivity and accuracy of aligners

Data set	Aligner	Mappability	Precision	Sensitivity	Accuracy
With 0% error	BiSpark†	0.9569	1.0	0.9569	0.9569
	Bismark	0.9454	1.0	0.9454	0.9454
	Bison	0.8030	0.6090	0.7129	0.4891
With 1% error	BiSpark†	0.9494	0.9892	0.9489	0.9392
	Bismark	0.9440	0.9961	0.9438	0.9403
	Bison	0.8297	0.5812	0.7391	0.4823
With 2% error	BiSpark†	0.9422	0.9800	0.9411	0.9234
	Bismark	0.9182	0.9862	0.9171	0.9055
	Bison	0.8315	0.5729	0.7387	0.4763

^†^The results from both BiSpark-plain and balance are denoted as BiSpark because the difference is only in the part where data is distributed, which means the results of two versions are always same

### Scalability evaluation to data size

We compared the scalability to data size by increasing input data size while cluster size remains unchanged. All real dataset in Table 1 were used and 20-cluster was used to execute CloudAligner, Bison, and the *BiSpark* while a single server was used to execute Bismark. Bismark supports parallel computing with a multicore option. However, there is no specific formulation of how many cores Bismark uses while execute Bismark with the multicore option. Instead, the user documentation of Bismark described that 4 multicore option would probably use 20 cores without any specific formulation. Therefore, we used 5 multicore option for safe comparison, even though 5 multicore option would use more than 21 cores.

**Fig. 2:**
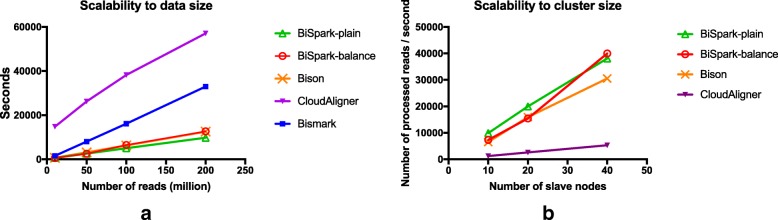
Comparison between the *BiSpark* and other bisulfite-treated aligners. In the performance test, the *BiSpark* outperforms all other aligners in terms of (**a**) scalability to data size and (**b**) cluster size

The performance evaluation result of each aligner in terms of scalability to data size is depicted in Fig. 2a. From the result, we could compare two evaluation points; one is a performance of speed itself inferred from *y*-axis value of each aligner measured in seconds. The other one is scalability to the number of reads inferred from the gradient of lines of each aligner. The scalability to the number of reads is getting more important in alignment process as the recent trend of sequencing depth becomes deeper resulting in large volumes of data.

The result showed that both versions of *BiSpark* outperform other aligners for both evaluation points. The estimated aligning time over the 10M reads data showed that *BiSpark*-plain only took 617 s and this is around more than 20 times faster than CloudAligner that took 14,783 s. This performance difference got higher when the larger volume of data set used. During the further evaluation though the data size increasing from 10M reads to 200M reads, the aligning time of Bismark was steeply increased from 1551 s to 32,972 s which means *BiSpark*-plain is around 2.5 times faster than Bismark on 10M reads and 3.5 times faster on 200M reads. That is, the more reads to be processed, the faster *BiSpark* is. From the comparison result with recently introduced Bison, the *BiSpark*-plain achieved around 22% performance improvement on 200M reads.

### Scalability evaluation to cluster size

We also compared the scalability to cluster size by increasing the number of slave nodes while data size remains unchanged. The dataset which consists of 100 million reads (16GB) was used as input and Bismark was excluded for this experiment since the experiment was done on the cluster.

The evaluation result of aligners which are able to be executed on the cluster is depicted in Fig. 2b. Unlike Fig. 2a, the *y*-axis of Fig. 2b is the number of processed reads per second, interpreted as throughput. We used this measurement since it is easier to visualize scalability by direct proportion curve than inverse proportion curve. The throughput which is inverse proportional to the performance of speed is inferred from *y* value of the plot while how well the aligner can scale up (out) is measured by the gradient of the plot where steeper gradient signifies better scalability.

We observed consistent result with the previous experiment for throughput analysis as the *BiSpark* showed the best throughput for all 10, 20 and 40 number of slave nodes, followed by Bison and CloudAligner. Also, the *BiSpark* scales up better than other aligners, which represents that the aligning module implemented within the *BiSpark* algorithm is highly parallelized and optimized. The *BiSpark*-balance showed relatively less throughput than *BiSpark*-plain for the cluster of 10 and 20 nodes but showed better throughput for the cluster of 40 nodes.

## Conclusions

We developed *BiSpark*, a highly parallelized Spark-based bisulfite-treated sequence aligner. The *BiSpark* not only shows the fastest speed for any size of the dataset with any size of the cluster but also shows the best scalability to both data size and cluster size. In addition, *BiSpark* improves practical usabilities that existing tools do not support. CloudAligner can only align sequencing reads to the single chromosome of reference genome per single execution. Bison has a restriction on cluster size and requires data to be manually distributed to all computing nodes before executing. The *BiSpark* alleviates these inconveniences by utilizing combination of the Spark framework over the HDFS.

We also developed *BiSpark*-balance which re-partitions RDDs in balance with additional shuffling. Since load balancing and shuffling are a trade-off in terms of the speed, it is hard to conclude theoretically whether the performance would be improved or not. Empirical results from our experiment showed that *BiSpark*-balance scaled well to data size but was generally slower than *BiSpark*-plain. However, *BiSpark*-balance showed better throughput when cluster size increased. The reason *BiSpark*-balance works faster for big cluster might be that the more nodes should wait for the slowest node as cluster size increases. In this case, re-partition can accelerate the aligning process even with the time-consuming shuffling operation since the throughput of the slowest node would be much more improved.

In this study, we newly implemented a bisulfite-treated sequence aligner over the distributed Apache Spark framework. We believe that by using the *BiSpark*, the burden of sequencing data analysis on bisulfite-treated methylome data could be significantly decreased and thus it allows large-scale epigenetic studies especially related with DNA methylation.

## Abbreviations

CPU: Central processing unit
SAM: Sequence alignment map
SNP: Single nucleotide polymorphism
